# Advancing kidney transplantation in black patients: a genetics-based and personalized approach under NICE, KDIGO, and ERBP guidelines

**DOI:** 10.1080/0886022X.2024.2438856

**Published:** 2024-12-15

**Authors:** Hatem Ali, Wisit Cheungpasitporn, Franco H. Cabeza Rivera, Yahya Makkeyah, Shafi Malik, Ákos G. Pethő, Pradeep Vaitla, Tibor Fülöp

**Affiliations:** a Renal Department, University Hospitals of North Midlands, Stoke-on-Trent, UK; b Division of Nephrology and Hypertension, Department of Medicine, Mayo Clinic, Rochester, MN, USA; c Katz Family Division of Nephrology and Hypertension, University of Miami Miller School of Medicine, Miami, FL, USA; d Kidney Department, University Hospitals of Leicester, Leicester, UK; e Kidney Department, University Hospitals of Birmingham, Birmingham, UK; f Department of Internal Medicine and Oncology, Faculty of Medicine, Semmelweis University, Budapest, Hungary; g Division of Nephrology, University of Mississippi Medical Center, Jackson, MS, USA; h Medical Services, Ralph H. Johnson VA Medical Center, Charleston, SC, USA; i Department of Medicine, Division of Nephrology, Medical University of South Carolina, Charleston, SC, USA

**Keywords:** Black ethnicity, donor-specific antibodies, induction therapy, kidney transplantation, kidney disease

## Abstract

Induction therapy is a critical component of renal transplantation, aimed at reducing delayed graft function (DGF) and improving graft survival. This review assesses the impact of leading large national and international guidelines: National Institute for Health and Care Excellence (NICE), Kidney Disease: Improving Global Outcomes (KDIGO), and European Renal Best Practice (ERBP) propositions, focusing on their applicability to high-risk groups, specifically, on Black patients and those with donor-specific antibodies (DSAs). While NICE guidelines provide a standardized approach favoring basiliximab, concerns arise regarding their suitability for high-risk patients, who may benefit more from potent lymphocyte-depleting agents. KDIGO and ERBP guidelines advocate for personalized approaches, emphasizing genetic diversity and specific patient profiles to tailor immunosuppressive regimens effectively. This review advocates for a paradigm shift toward personalized induction therapy, integrating genetic insights to improve clinical outcomes and address health disparities. By tailoring induction therapies to the genetic and immunological profiles of transplant recipients, healthcare providers can enhance transplant success and ensure equitable healthcare for diverse populations. This approach underscores the importance of personalized medicine in achieving optimal outcomes in renal transplantation. This concern is of particular importance to Black individuals due to the specific genetic markers and health profiles relevant to this group, while recognizing the current gap in data regarding other ethnicities.

## Introduction

Induction therapy, a critical component in renal transplantation, involves the use of immunosuppressive agents such as interleukin-2 receptor antagonists (IL-2RA) or lymphocyte-depleting agents. Historically, the use of induction therapy has evolved significantly, with a growing body of evidence supporting its role in reducing delayed graft function (DGF)—a common complication following transplantation that can negatively impact graft survival. DGF, characterized by the need for dialysis within the first week post-transplantation, is associated with increased morbidity, mortality, and healthcare costs [1, 2]. Several retrospective studies have demonstrated the efficacy of induction therapy in mitigating the incidence of DGF, particularly in recipients of deceased donor kidneys (DCD), who are at higher risk of this complication [3–6].

Despite the recognized benefits of induction therapy, the choice of the specific agent remains a subject of debate within the transplant community. The lack of consensus stems from the variability in patient and donor characteristics, as well as the differential impact of various immunosuppressive agents on patient outcomes. This variability necessitates a tailored approach to induction therapy, considering factors such as the immunological risk of the recipient, the type of donor organ (living versus deceased donor), and the presence of donor-specific antibodies (DSAs).

The National Institute for Health and Care Excellence (NICE) guidelines of 2017 represented a pivotal moment in the standardization of care for renal transplant recipients in the United Kingdom. These guidelines recommended the use of basiliximab, an IL-2RA, as the preferred agent for induction therapy across a broad spectrum of transplant scenarios. This recommendation was based on a comprehensive review of the available evidence, aiming to streamline care and ensure the best possible outcomes for transplant recipients [7]. However, the broad application of these guidelines has raised concerns among transplant specialists, particularly regarding their applicability to patients with unique or high-risk profiles. For instance, patients of Black ethnicity, those with preexisting DSAs, or those receiving a second or subsequent transplant may exhibit a heightened immunological response, increasing the risk of acute rejection and graft loss. These patient populations may benefit from more potent induction therapies, such as lymphocyte-depleting agents, to adequately suppress the immune response and improve graft survival.

In contrast, the Kidney Disease: Improving Global Outcomes (KDIGO) international guidelines offer a more detailed and flexible approach to induction therapy. KDIGO recommends IL-2RAs like basiliximab for most patients but advocates for the use of more potent lymphocyte-depleting agents, such as antithymocyte globulin (ATG) or alemtuzumab, in high-risk groups including Black patients and those with DSAs. KDIGO emphasizes personalized medicine, considering genetic diversity and specific patient profiles to tailor immunosuppressive regimens effectively [4].

Similarly, the European Renal Best Practice (ERBP) guidelines align closely with KDIGO, advocating for personalized approaches to induction therapy. They recognize the unique challenges faced by high-risk populations and recommend potent induction agents and tailored immunosuppressive regimens based on individual risk factors. The ERBP guidelines support continuous monitoring and adjustments to therapy, emphasizing the importance of genetic and immunological assessments [8].

However, a significant limitation across these guidelines is the unclear definition of high-risk groups, including Black ethnicity and patients with DSAs, and the implications this has for tailoring induction therapies. Black patients often face higher immunological risks due to factors such as HLA diversity and faster metabolism of immunosuppressive drugs. The lack of a precise, genetics-based definition of Black ethnicity and specific criteria for high-risk groups, like the presence of donor specific antibodies, in these guidelines fails to address the complexities and needs of these populations adequately.

In light of these considerations, this review aims to critically assess the current NICE, KDIGO, and ERBP guidelines for induction therapy in renal transplantation, with a particular focus on their applicability to diverse patient populations. By reviewing the latest evidence on the efficacy of various induction therapy agents and examining the outcomes of personalized approaches, we seek to identify potential areas for improvement in these guidelines. Our ultimate goal is to contribute to the development of a more nuanced and effective framework for induction therapy, one that recognizes the complexity of renal transplantation and the diverse needs of transplant recipients.

## Methodology for literature search

Our mini-review is focused and targeted, aiming to provide a succinct overview of induction therapy in renal transplantation, particularly exploring genetic considerations for Black patients. Here is how we approached the literature:

1. Literature Search Strategy: We selectively searched PubMed and MEDLINE for recent articles published within the last decade, using keywords like ‘renal transplantation,’ ‘induction therapy,’ and ‘genetic markers.’

2. Selection Criteria: We included articles based on relevance to our topic, prioritizing high-quality, peer-reviewed sources that contribute meaningfully to the discussion on genetic diversity and personalized medicine in transplantation.

3. Data Synthesis: The narrative synthesis allowed us to integrate and discuss significant findings related to genetic markers and their implications in clinical settings, emphasizing advancements and insights pertinent to Black patients.

This focused approach ensures our mini-review remains concise yet informative, providing valuable insights into a specialized area of renal transplantation.

### Evaluation of NICE, KDIGO, and ERBP guidelines

To comprehensively assess the applicability of induction therapy guidelines to diverse patient populations, we will evaluate the strengths and limitations of the NICE, KDIGO, and ERBP guidelines. This evaluation will focus on their recommendations for high-risk groups, including Black patients and those with DSAs, and the flexibility of these guidelines in accommodating individual patient needs.

### NICE guidelines

The National Institute for Health and Care Excellence (NICE) guidelines of 2017 represented a pivotal moment in the standardization of care for renal transplant recipients in the United Kingdom ^7^. These guidelines recommended the use of basiliximab, an IL-2RA, as the preferred agent for induction therapy across a broad spectrum of transplant scenarios. This recommendation was based on a comprehensive review of the available evidence at that time, aiming to streamline care and ensure the best possible outcomes for transplant recipients. However, the broad application of these guidelines has raised concerns among transplant specialists, particularly regarding their applicability to patients with unique or high-risk profiles. For instance, patients of black ethnicity, those with preexisting DSAs, or those receiving a second or subsequent transplant may exhibit a heightened immunological response, increasing the risk of acute rejection and graft loss. These patient populations may benefit from a more potent induction therapy, such as lymphocyte-depleting agents, to adequately suppress the immune response and improve graft survival.

The NICE guidelines’ stance against recommending anti-thymocyte globulin (ATG)) as a standard induction therapy is rooted in a comprehensive evaluation of risk profiles for both kidney donors and recipients. These guidelines underscore that immunological risk is influenced by a constellation of factors, including panel-reactive antibody levels. The committee’s deliberations, informed by studies like Brennan’s in 2006, highlighted notable differences in outcomes such as acute rejection and CMV infection rates when comparing basiliximab, an IL-2RA and rabbit-derived (r)-ATG [7]. This nuanced decision-making process, balancing empirical evidence against the practicalities of clinical experiences, led to the conclusion that r-ATG, despite its efficacy, did not demonstrate enough advantage in terms of clinical outcomes or cost-effectiveness to warrant a broad recommendation, particularly for patients with high immunological risk [7–10]. This decision reflects a cautious and evidence-based approach but also raises questions about the flexibility of these guidelines in accommodating diverse patient needs (Table 1).

**Table 1.** The key aspects and points derived from the evaluation of NICE guidelines, ATG induction therapy, and considerations for personalized induction therapy in renal transplantation.

Aspect	Key points
NICE guidelines & ATG Therapy	NICE guidelines recommend basiliximab over ATG for most patients, based on a comprehensive evaluation including risk profiles and outcomes like acute rejection and CMV infection rates.
Advocacy for personalized induction therapy	There’s a call for more tailored immunosuppression regimens that consider the unique immunological profiles of recipients, to improve outcomes and reduce adverse effects.
Black ethnicity consideration	Black patients face higher immunological risks, potentially requiring more potent induction therapy. The debate on cytolytic vs. IL-2RA induction therapies is ongoing.
Comparative efficacy studies	Studies have shown varying outcomes for different induction therapies among high-risk groups, including black patients, with a general trend toward better outcomes with cytolytic induction.
Presence of donor specific antibodies	Low-level DSAs detected by Luminex, even with negative flow cytometry, may not significantly increase risk, questioning the necessity of avoiding PLNF transplants.
Factors increasing risk in black patients	Higher risk of rejection in Black patients may be due to genetic differences, immunological response, medication metabolism, and antibody development.
Racial disparities in CKD	The absence of race-specific recommendations in major guidelines fails to address the needs of racially diverse populations, highlighting the need for individualized care protocols.
Evaluation criteria for DSAs	Thresholds for significance, specificity of antibodies, and dynamics of antibodies are crucial in evaluating the risk of rejection and tailoring induction therapy.

Abbreviations: ATG: Anti-Thymocyte Globulin; CMV: Cytomegalovirus; CKD: Chronic Kidney Disease; DCD: Deceased Donor Kidney Transplants; DSA: Donor-Specific Antibodies; IL-2RA: Interleukin-2 Receptor Antagonists; NICE: National Institute for Health and Care Excellence; PLNF: Positive Luminex and Negative Flow.

### KDIGO guidelines

The KDIGO (Kidney Disease: Improving Global Outcomes) guidelines are globally recognized and offer a more detailed and flexible approach to induction therapy. These guidelines recommend interleukin-2 receptor antagonists (IL-2RAs) like basiliximab for most patients, but strongly advocate for the use of more potent lymphocyte-depleting agents, such as anti-thymocyte globulin (ATG) or alemtuzumab, in high-risk groups, including Black patients and those with donor-specific antibodies (DSAs). KDIGO emphasizes personalized medicine, considering genetic diversity and specific patient profiles to tailor immunosuppressive regimens effectively [4].

KDIGO guidelines are considered more comprehensive and are developed through a rigorous scientific process involving global experts. They provide detailed recommendations based on the latest evidence, ensuring that they address diverse patient needs and scenarios, making them particularly crucial in the context of high-risk groups and complex cases. This personalized approach aligns with the growing body of evidence supporting the need for tailored immunosuppressive regimens based on individual patient profiles and genetic backgrounds.

### European renal best practice (ERBP) guidelines

The European Renal Best Practice (ERBP) guidelines align closely with KDIGO in advocating for personalized approaches to induction therapy. They recognize the unique challenges faced by high-risk populations and recommend potent induction agents and tailored immunosuppressive regimens based on individual risk factors. The ERBP guidelines support continuous monitoring and adjustments to therapy, emphasizing the importance of genetic and immunological assessments [8].

The KDIGO and the ERBP guidelines were based on a previous meta-analysis involving 16 studies and 2,211 participants that compared the efficacy of IL-2RA with ATG [11]. The studies primarily included patients at low immunological risk, characterized by their first transplant and absence of anti-human leukocyte antigen (HLA) antibodies. The analysis found no significant difference in graft loss or clinically diagnosed acute rejection between the two therapies. However, ATG showed a slight advantage in reducing biopsy-proven acute rejection at one year, albeit with increased risks. Specifically, ATG therapy was associated with a 75% higher incidence of malignancies, predominantly post-transplant lymphoproliferative disorder (PTLD), and a 32% higher incidence of cytomegalovirus (CMV) disease. Patients receiving ATG also experienced more frequent adverse reactions, including fever, cytokine release syndrome, and leukopenia, though not thrombocytopenia. The outcomes were consistent regardless of the use of cyclosporine or tacrolimus, azathioprine or mycophenolic acid (MPA), and the baseline risk for acute rejection. No differences were noted between the use of equine versus rabbit ATG. Thus, for low-risk patients, IL-2RA was generally preferred over ATG due to its more favorable safety profile.

In contrast, the scenario changes for high-risk transplant recipients. Two prospective randomized trials, one focusing on patients at high risk for delayed graft function and the other on those with high immunological risk, demonstrated that ATG significantly reduced the risk of acute rejection compared to IL-2RA [9, 12]. As a result, the European Renal Best Practice (ERBP) guidelines support the use of ATG for high-risk patients. High-risk factors include high panel reactive antibodies, transplantation across a donor-specific antibody barrier, multiple previous transplants, previous graft loss due to immunological reasons, 5–6 HLA mismatches, donation after cardiac death, and cold ischemia time exceeding 24 h.

It is important to note that the increased risk of CMV infection observed with ATG therapy in this meta-analysis was recorded during a period when CMV treatment and prophylaxis were not as advanced as they are today. At that time, valganciclovir, a more effective antiviral agent for CMV prophylaxis and treatment, was not available, potentially contributing to the higher incidence of CMV-related complications.

### FDA approval for use of rabbit-ATG

The U.S. Food and Drug Administration’s (FDA) recent approval of rabbit anti-thymocyte globulin (rATG) for the prophylaxis and treatment of acute rejection in kidney transplant patients underscores a significant regulatory approach toward the integration of retrospective data in drug approvals [13]. Historically, the FDA has emphasized the importance of prospective randomized controlled trials (RCTs) for establishing drug efficacy and safety. However, the approval based on retrospective analyses indicates a shift toward a more flexible, evidence-based approach that considers real-world effectiveness. This decision reflects an understanding that retrospective studies can offer extensive insights into long-term outcomes and real-world usage that RCTs might not fully capture, especially in scenarios where conducting prospective studies is challenging or not feasible. The inclusion of retrospective data allows for a broader evaluation of patient outcomes across diverse populations and clinical settings, providing a more comprehensive assessment of the treatment’s practical benefits and risks. Such regulatory openness is crucial for advancing therapeutic options in fields like transplantation, where evolving clinical practices and varied patient demographics demand dynamic evidence assessment.

Table 2 shows a comparison between all the three guidelines.

**Table 2.** Comparison between the recommendations of the three guidelines.

Guidelines	Induction recommendation	Summary statements	Major references
NICE	Basiliximab preferred	Recommends basiliximab for most patients due to efficacy and safety profiles. Less favorable toward rATG except in high immunological risk cases.	Brennan et al. (2006); NICE (2017)
KDIGO	Tailored approach	Suggests IL-2RAs like basiliximab generally, but recommends potent agents like ATG for high-risk groups, including Black patients and those with DSAs.	KDIGO (2009); Pilch et al. (2014)
ERBP	Personalized therapy	Aligns with KDIGO to recommend more potent induction agents for high-risk groups and emphasizes continuous monitoring and adjustments based on genetic and immunological assessments.	Heemann et al. (2011); Webster et al. (2010); Brennan et al. (2006)
FDA	rATG for prophylaxis and treatment of acute rejection	Approved based on retrospective data, highlighting the utility and safety of rATG in conjunction with other immunosuppressants for both prophylaxis and treatment of acute rejection in kidney transplant recipients.	Latest package insert

#### Defining Black ethnicity

While evaluating the guidelines provides insight into their strengths and limitations, a crucial aspect of tailoring induction therapy lies in the precise definition of high-risk groups, such as Black ethnicity [12–14]. Defining Black ethnicity based on genetics involves identifying specific genetic markers commonly found in individuals of African descent [14]. This genetic approach offers a more precise understanding of the diversity within the Black population, enabling tailored medical treatments to improve clinical outcomes effectively.

#### Genetic markers

##### Human Leukocyte Antigen (HLA) Alleles

Human Leukocyte Antigen (HLA) genes are crucial components of the immune system, playing a significant role in the body’s ability to recognize foreign tissues. Certain HLA alleles are more prevalent among individuals of African descent, contributing to a higher degree of genetic diversity within this population. This diversity can complicate donor-recipient matching and increase the risk of transplant rejection. Studies indicate that Black patients often exhibit a broader range of HLA alleles, which can significantly influence compatibility and rejection risks in kidney transplantation [14–17].

##### CYP3A5 gene

The CYP3A5 gene is essential for the metabolism of various drugs, including immunosuppressive medications used in transplantation. A common variant, CYP3A5*1, is more frequently observed in individuals of African descent. This variant results in higher enzymatic activity, leading to faster metabolism of immunosuppressive drugs such as tacrolimus. Consequently, Black patients often require higher doses or alternative medications to achieve therapeutic levels, underscoring the necessity for personalized dosing regimens based on genetic testing [18].

##### APOL1 gene

Variants in the APOL1 gene, particularly the G1 and G2 alleles, are strongly associated with an increased risk of kidney disease and are more prevalent in people of African ancestry. These genetic variants are linked to higher susceptibility to conditions such as focal segmental glomerulosclerosis (FSGS) and HIV-associated nephropathy (HIVAN). The presence of APOL1 risk alleles can adversely affect kidney transplant outcomes, making genetic screening crucial for this population [19].

Table 3 shows the key characteristics of these markers and its implications for induction therapy.

**Table 3.** Genetic markers and their implications for induction therapy in black patients.

Genetic marker	Prevalence in Black patients	Impact on kidney transplantation	Implications for induction therapy
HLA alleles	Higher diversity, more frequent mismatches	Increased risk of transplant rejection due to poor matching	May require more potent induction therapy to prevent rejection
CYP3A5	Common CYP3A5*1 variant leads to higher enzyme activity	Faster drug metabolism, especially tacrolimus	Higher doses or alternative medications may be needed to achieve therapeutic levels
APOL1	High prevalence of risk alleles G1 and G2	Associated with increased risk of chronic kidney disease and poorer transplant outcomes	Genetic screening recommended; may influence donor selection and post-transplant management

#### Genetic testing and population studies

##### Genome-wide association studies (GWAS)

Genome-wide association studies (GWAS) are pivotal in identifying genetic variations associated with specific traits or diseases across diverse populations. By including individuals from various ethnic backgrounds, GWAS can pinpoint genetic markers significant in Black populations. These studies enhance our understanding of the genetic underpinnings of kidney disease and transplant outcomes, facilitating the development of more effective, personalized treatment strategies [20].

##### Ancestry informative markers (AIMs)

Ancestry informative markers (AIMs) are genetic markers that display considerable frequency differences between populations from different continents. AIMs are utilized to estimate an individual’s ancestral background, offering insights into genetic diversity. This information is invaluable in understanding how ancestral genetic variations influence disease susceptibility and treatment responses in Black populations [21, 22].

##### Biogeographical ancestry (BGA) testing

Biogeographical Ancestry (BGA) testing involves analyzing specific genetic markers across the genome to determine the proportion of an individual’s ancestry from various geographical regions. BGA testing can elucidate the genetic contributions from African ancestors in individuals of mixed heritage. This genetic insight is crucial for accurately assessing risks and tailoring medical treatments in kidney transplantation [21, 22].

#### Implementation in clinical practice

##### Tailored medical treatments

Utilizing genetic testing to identify relevant markers allows healthcare providers to tailor immunosuppressive therapies more effectively. For instance, adjusting drug dosages based on CYP3A5 genotyping can optimize the management of immunosuppressive medications in Black transplant recipients, ensuring therapeutic efficacy and minimizing adverse effects [23].

##### Reducing health disparities

A genetics-based definition of Black ethnicity can address health disparities by providing more precise medical care. This approach ensures that high-risk individuals receive appropriate monitoring and treatment adjustments, thereby improving clinical outcomes and promoting equity in healthcare.

Incorporating genetic insights into the definition of Black ethnicity can significantly enhance the precision and effectiveness of medical treatments in kidney transplantation. By recognizing the genetic diversity within the Black population and tailoring induction therapies accordingly, we can improve transplant outcomes and reduce health disparities. This approach underscores the importance of personalized medicine in achieving optimal healthcare for all patients [24, 25].

#### Advocacy for personalized induction therapy

**Figure 1.**
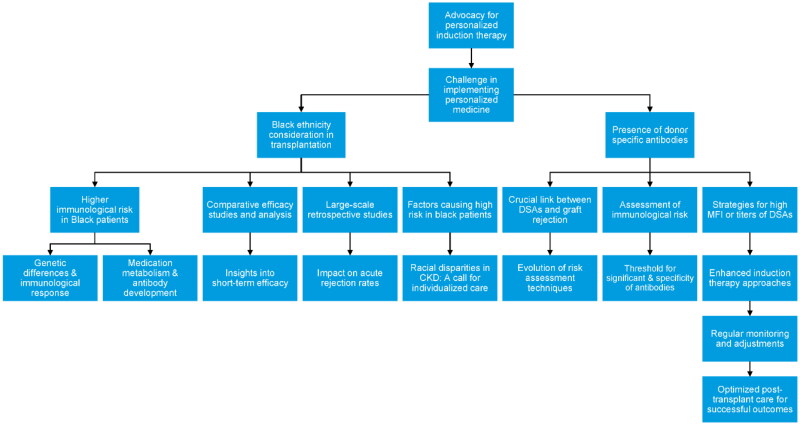
Advocacy for personalized therapy.

Building on the understanding that genetic diversity significantly influences transplant outcomes, it becomes clear that a one-size-fits-all approach is inadequate. Personalized induction therapy, which takes into account the unique genetic and immunological profiles of each patient, is essential for optimizing transplant success and minimizing adverse effects. We advocate for a reconsideration of these generalized recommendations. The landscape of immunosuppressive therapy has seen significant advancements in recent years, with the introduction of novel agents and improved diagnostic tools for early detection of antibody-mediated injury. These developments present a compelling case for adopting more tailored immunosuppression regimens. We propose that integrating personalized medicine into kidney transplantation protocols—by acknowledging the distinct immunological profiles of recipients—could markedly enhance patient outcomes through the reduction of adverse effects and graft loss. This approach, however, confronts challenges due to the current global reliance on rigid, protocol-driven strategies with minimal variation, highlighting the imperative for a paradigm shift toward more individualized care. Critical factors, such as the patient’s ethnicity—specifically Black ethnicity—and the presence of donor-specific antibodies, play a significant role in elevating the risk of adverse outcomes in kidney transplantation, further underscoring the necessity of this tailored approach (Figure 1).

## Black ethnicity

### The Black ethnicity consideration in transplantation

In the UK, where individuals of black ethnicity constitute approximately 9% of deceased donor transplants and 11% of the waiting list, there is a recognized higher immunological risk associated with transplantation [24, 25]. This categorization stems from a range of biological and socioeconomic factors that uniquely affect this demographic. The current debate on the efficacy of cytolytic versus IL-2RA induction therapies within this group is hampered by limited study sizes and follow-up periods, which do not adequately capture long-term patient or graft survival outcomes. Furthermore, the underrepresentation of black individuals in RCTs adds another layer of complexity to this issue, making it challenging to draw definitive conclusions about the most effective induction therapy in this population. Similarly, in the USA, the black ethnicity patients with chronic kidney disease is 35% [26–28].

### Comparative efficacy studies and analysis

Studies like Brennan et al. and Pilch et al. have provided insights into the short-term efficacy of different antibody induction therapies in high-risk kidney transplant recipients, including black patients [9, 12]. However, these studies lacked the statistical power and longitudinal follow-up necessary to assess long-term outcomes such as graft loss and patient survival, particularly among black patients. In contrast, analyses using national registry data have been able to offer more conclusive evidence regarding the impact of cytolytic induction on long-term outcomes, suggesting a reduction in acute rejection rates within this demographic.

### Large-scale retrospective studies

Large-scale retrospective studies, such as those conducted by Patlolla et al. and Jindal et al. have leveraged transplant registry data to compare the effectiveness of different induction therapies [29, 30]. These studies have generally found that cytolytic induction therapies can decrease acute rejection rates. However, they also noted that these benefits did not significantly translate into improved graft survival rates when compared to IL-2RA induction therapy. Jindal’s analysis, focusing on African American patients, observed a trend toward reduced graft loss with rATG, although this finding was not statistically significant.

The study by Taber et al. in 2017 stands as a significant contribution to this field [31]. Utilizing a comprehensive national cohort, the research assessed outcomes like acute rejection, graft loss, and mortality among African American kidney transplant recipients. The findings were revealing: cytolytic induction therapy notably reduced the risk of acute rejection, graft loss, and death, particularly among patients who were sensitized, had public insurance, experienced delayed graft function, or underwent steroid withdrawal. This study underscores the potential benefits of cytolytic induction therapy in a specific, high-risk demographic, providing a strong argument for the personalized approach in induction therapy.

## Factors causing black patients a high risk transplant

The higher risk of kidney transplant rejection in Black patients can be attributed to several factors:

1. Genetic Differences: Black patients may have certain genetic variations that affect their immune response, leading to a higher likelihood of transplant rejection. One such example is the higher frequency of certain Human Leukocyte Antigen (HLA) mismatches [32, 33].
2. Immunological Response: Research suggests that Black patients might have a more robust immunological response to transplanted organs, increasing the risk of rejection [34, 35].
3. Medication Metabolism: Differences in the metabolism of immunosuppressive drugs, which are crucial in preventing rejection, might also play a role. For example, Black patients often require higher doses of certain medications to achieve the same therapeutic effect [36, 37].
4. Antibody Development: There’s evidence suggesting that Black patients are more likely to develop certain antibodies against the transplant, which can lead to rejection [34, 38].
5. Research and Data Limitations: Historically, clinical trials and medical research have underrepresented Black individuals, leading to gaps in knowledge and potentially less effective treatment protocols for this group [14].

### Racial disparities in CKD: a call for individualized care in transplant protocols

In evaluating global practices for induction therapy in kidney transplantation, it’s imperative to note that prominent guidelines like those from KDIGO (USA) and the European Association of Urology (EAU) do not incorporate race-specific recommendations [4, 6]. This omission underscores a significant gap in addressing the nuanced needs of racially diverse populations. In the United States, where the black population constitutes approximately 13.6% of the total, translating to about 45.4 million people, they represent a disproportionately high 30% of individuals with End-Stage Kidney Disease (ESKD), according to the American Kidney Fund [39]. The Centers for Disease Control and Prevention (CDC) further elucidates this disparity, indicating that non-Hispanic Black adults have a CKD prevalence of about 20%, significantly higher than their non-Hispanic White and Hispanic counterparts [40]. This stark absence of racial considerations in these guidelines contrasts with the demographic realities and the disproportionate impact of CKD on the black community. This review advocates for a reevaluation of such guidelines to include or at least emphasize the necessity for individualized induction protocols, incorporating race-based insights to offer a more nuanced approach to treatment, potentially improving outcomes for vulnerable populations and ensuring equity in transplantation medicine.

### Addressing racial disparities in transplant outcomes: a comparative view between the UK and USA

While advances in kidney transplantation have significantly improved overall patient outcomes, disparities persist, particularly among patients of Black ethnicity in both the UK and the USA. These disparities are not just clinical but are deeply rooted in a complex interplay of genetic, socio-economic, and systemic factors that vary significantly between these two countries.

In the United States, Black patients undergoing kidney transplantation generally experience worse outcomes compared to their White counterparts [41]. This discrepancy is largely attributed to a higher prevalence of certain genetic markers such as APOL1, which are associated with an increased risk of kidney disease and poorer transplant outcomes. Moreover, socio-economic factors, including access to healthcare, insurance coverage, and disparities in pre- and post-transplant care, further exacerbate these outcomes. The impact of these factors is profound, with Black patients facing higher rates of graft rejection and lower survival rates post-transplantation.

Conversely, in the UK, where healthcare is universally accessible *via* the National Health Service (NHS), disparities still exist but manifest differently. Access to care is less of a barrier; however, socio-economic status and a lack of culturally competent healthcare practices still influence outcomes negatively. Furthermore, the genetic risks, while similar, are influenced by a more diverse Black population, which includes individuals of African and Caribbean descent, each with distinct health profiles and challenges.

These disparities highlight the critical need for tailored approaches in the management of kidney transplant patients. Current guidelines often fail to address the unique needs of racially diverse populations adequately. For instance, induction therapies that do not consider genetic susceptibility or potential socio-economic barriers to follow-up care may not be as effective in mitigating the risk of rejection or prolonging graft survival in these populations.

### Presence of donor-specific antibodies

Patel and Terasaki’s pioneering work in 1969 established a crucial link between the presence of pre-formed DSAs in recipients of organ transplants and an increased likelihood of graft rejection [42]. This foundational study shed light on the varying degrees of immunological risk associated with HLA-incompatible kidney transplants. It has become clear that not every recipient can afford to wait for an HLA-compatible donor, and for those receiving HLA-incompatible transplants, survival rates have shown a marked improvement compared to those who continue on dialysis, as indicated in subsequent research [43]. The assessment of immunological risk has evolved, with a positive complement-dependent cytotoxic crossmatch (CDCXM) indicating high risk and a negative flow cytometry crossmatch (FCXM) suggesting lower risk. The introduction of Luminex single-antigen bead technology has further refined our understanding of HLA compatibility. This technology has revealed that recipients previously deemed HLA-compatible based on negative FCXM results may still possess undetected DSAs, identifiable only through Luminex testing. A recent meta-analysis, which encompassed all available studies on kidney transplantation in patients with positive Luminex and negative flow cytometry results, determined that the exclusion of transplants based on low-level DSAs, detected solely by Luminex (referred to as PLNF transplants), may not be justified, particularly in light of the current kidney donor shortage [44]. This systematic review highlighted that, in the majority of reviewed studies, the induction therapies applied were predominantly ATG and alemtuzumab. Furthermore, the British Transplantation Society’s guidelines on ‘The Detection and Characterization of Clinically Relevant Antibodies in Allotransplantation’ advise considering enhanced immunosuppression and vigilant post-transplant immunological monitoring for recipients in the presence of low levels of DSAs at the time of transplantation [45].

In the context of kidney transplantation, when considering induction therapy for a patient with a positive Luminex and a negative flow cytometry, the titers and Mean Fluorescence Intensity (MFI) of donor-specific antibodies (DSAs) detected by the Luminex assay are crucial factors. In evaluating these factors, transplant teams consider:

- Threshold for Significance: Each transplant center may have different thresholds for what they consider a clinically significant MFI or titer level. These thresholds are based on clinical experience and research.
- Specificity of Antibodies: The specific target of the DSAs (e.g., HLA Class I or Class II antigens) can also influence the risk assessment and induction therapy.
- Dynamics of Antibodies: Changes in MFI or titers over time can provide insights into the risk of rejection. Increasing levels might indicate a higher risk and may influence the choice of more aggressive induction therapy.

In cases where kidney transplant recipients present with high MFI or high titers of DSAs, despite having a negative flow cytometry, a more intensive induction therapy approach is recommended. This enhanced strategy might encompass the administration of potent immunosuppressive agents such as ATG and Rituximab. Additionally, desensitization treatments like plasmapheresis and intravenous immunoglobulin (IVIG) may be employed to lower antibody levels, aiming to decrease the likelihood of both acute and chronic rejection episodes. These measures are crucial for mitigating immunological risks and improving the transplant’s success rate [42–45].

We recommend that the interpretation of immunological tests, including Luminex assays for DSAs, and the subsequent management plans should be highly personalized. This approach should take into account the comprehensive clinical context of each patient, including their transplant history and any additional risk factors present. It is crucial to engage in regular monitoring and to adjust treatment plans as necessary, based on the patient’s response to therapy and the ongoing function of the graft [42–45]. This tailored strategy ensures that post-transplant care is optimized for each individual, thereby enhancing the potential for successful outcomes and long-term graft survival.

#### Rebuttal: cost-effectiveness of rATG vs. Basiliximab in high-risk kidney transplant recipients

The UK’s National Institute for Health and Care Excellence (NICE) guidelines generally recommend basiliximab as the induction therapy for most kidney transplant patients. This recommendation is based on broad studies showing good efficacy combined with a favorable side effect profile and lower costs compared to other induction agents such as rATG (rabbit anti-thymocyte globulin). However, this one-size-fits-all approach may not suit all patient groups, particularly those at high immunological risk.

NICE guidelines advocate for basiliximab due to its cost-effectiveness in the general transplant population. These guidelines are shaped by data that predominantly reflect average-risk scenarios, where the prevention of acute rejection does not necessitate the powerful immunosuppression that rATG provides. Furthermore, NICE emphasizes the lower incidence of side effects and overall lower treatment costs associated with basiliximab.

Contrasting sharply with NICE’s stance, a detailed analysis in a German study published in ‘Transplant International’ presents compelling evidence favoring rATG in high-risk groups [36]. This study points out that while rATG is more expensive upfront, it significantly reduces the long-term incidence of graft rejection and other severe complications in patients who are at high immunological risk. These include recipients with prior transplant failures, high panel-reactive antibody levels, or those receiving a transplant from a deceased donor.

The German study’s economic model, which takes a long-term view of patient outcomes, suggests that the initial higher costs of rATG are offset by the reduced costs associated with treating graft failure and managing chronic rejection. The study quantifies this by demonstrating a lower total cost of care over a 10-year period, alongside an increase in quality-adjusted life years (QALYs), making rATG more cost-effective for this particular subgroup.

This brings to light a critical oversight in the NICE guidelines—the lack of differentiation between general and high-risk patient groups. The application of a uniform guideline across such a diverse patient base can lead to suboptimal outcomes for those at the highest risk. The German study advocates for a more nuanced approach, suggesting that the benefits of rATG in high-risk groups significantly outweigh its higher initial cost, particularly when considering long-term outcomes and overall healthcare expenditures.

Given the evidence presented, it is prudent to advocate for a revision of current NICE guidelines regarding induction therapy in kidney transplantation. Specifically, there is a strong case for the preferential use of rATG in high-risk kidney transplant recipients, where its higher efficacy at preventing severe complications can lead to greater overall cost savings and improved patient outcomes. Tailoring induction therapy to the risk profile of the recipient not only aligns with principles of personalized medicine but also ensures cost-effective healthcare delivery.

While this review provides a comprehensive analysis of induction therapy tailored to Black patients, it is important to note the limitation in the scope of data available. Current research predominantly addresses genetic and immunological profiles unique to Black individuals, which are not universally applicable to other ethnic groups. The absence of data for other races and ethnicities underscores a significant gap in the literature, highlighting an urgent need for expanded research to ensure inclusive healthcare practices that can cater to the diverse global population. Future investigations should focus on prospective, randomized controlled trials to validate the benefits of such individualized induction strategies (Table 4).

**Table 4.** Areas for future research in the field of induction therapy for kidney transplantation, driven by the need to improve patient outcomes, address disparities, and ensure cost-effectiveness within transplant medicine.

Study focus	Objective	Why these studies are needed
Prospective randomized trials on induction therapy	To validate the benefits of tailored induction strategies through controlled, prospective studies.	To provide high-quality evidence on the efficacy and safety of personalized induction therapies, facilitating the adoption of best practices in clinical settings.
Impact of personalized induction therapy	To assess patient outcomes when induction therapy is personalized based on immunological profiles.	To demonstrate how customizing therapy to individual patient needs can improve outcomes and reduce adverse effects, supporting the case for personalized medicine in kidney transplantation.
Long-term outcomes of PLNF transplants	To evaluate the long-term graft survival and patient mortality in PLNF transplant recipients.	To fill the knowledge gap on the impact of low-level DSAs on transplant success, potentially challenging current guidelines and practices regarding PLNF transplants.
Induction therapy efficacy in diverse racial groups	To determine the differential impact of induction therapy across various racial and ethnic groups.	To address racial disparities in transplantation outcomes by tailoring induction therapy to the specific needs of diverse populations, promoting equity in transplant care.
Cost-effectiveness of tailored induction protocols	To analyze the cost-effectiveness of personalized versus standard induction therapy protocols.	To evaluate the economic viability of personalized induction therapy, ensuring that the benefits justify any additional costs and supporting sustainable healthcare practices.
Comparative studies on induction agents	To compare the efficacy and safety of different induction agents in a head-to-head manner.	To provide clear guidance on the selection of induction agents based on comparative effectiveness, enhancing clinical decision-making.
Impact of genetic variations on transplant outcomes	To investigate how genetic variations among recipients affect the success rate of kidney transplants.	To understand the role of genetic factors in transplant outcomes, enabling more precise matching and treatment plans that could improve graft survival rates.
Efficacy of desensitization treatments	To measure the effectiveness of desensitization treatments like plasmapheresis and IVIG in reducing DSAs.	To assess the role of desensitization in managing high-risk transplants and improving outcomes, potentially expanding the pool of viable organs for transplantation.
Monitoring and adjustment strategies post-transplant	To develop and test the effectiveness of strategies for ongoing monitoring and treatment adjustment post-transplant.	To optimize post-transplant care through evidence-based monitoring and treatment adjustments, enhancing long-term patient and graft survival.

Abbreviations: DSAs: Donor-Specific Antibodies; IVIG: Intravenous Immunoglobulin; PLNF: Positive Luminex and Negative Flow.

## African countries and induction therapy

Data on induction therapy in African countries performing kidney transplants is sparse due to several challenges related to healthcare infrastructure, training, and research capabilities. Several factors contribute to this lack of data:

1. Infrastructure and Training: Many African countries are still in the nascent stages of establishing transplantation programs. For instance, Tanzania recently initiated its kidney transplantation services and has faced significant challenges, including the need for extensive training of healthcare personnel and setting up adequate facilities [46].
2. Limited Research and Publications: There is a scarcity of published research on kidney transplantation from African settings due to limited funding, lower numbers of transplants performed, and less emphasis on research. Most African countries with transplantation programs like South Africa, Nigeria, and Egypt have published some data, but these are limited and focus more on outcomes rather than specific induction protocols [47].
3. Regulatory and Ethical Frameworks: Many countries are still developing legal and ethical frameworks to manage and regulate transplantation, which also impacts the ability to conduct and publish research. For instance, specific guidelines and regulations are needed to ensure ethical practices in organ donation and transplantation [48].

Despite these challenges, some studies and reviews have been done. For example, a study from Tanzania highlighted the clinical profiles and outcomes at a national hospital, which included discussions on induction therapy used in the transplantation process [48].

However, these studies are often limited in scope and lack detailed comparative data on induction protocols, which are crucial for understanding best practices and improving outcomes.

Improving data collection and publication from African countries will require enhanced collaboration with international partners, increased investment in healthcare infrastructure, and a focus on training and retaining skilled healthcare professionals in nephrology and transplant medicine.

### Other induction therapies and their role in high-risk populations

Alemtuzumab, a potent lymphocyte-depleting agent, has shown promise in high-risk renal transplant populations, particularly in addressing racial disparities. A study by Smith et al. (2015) demonstrated that alemtuzumab induction significantly reduced graft failure rates among African American (AA) recipients, eliminating the racial disparity in outcomes between AA and white recipients. However, it is important to note that this study was retrospective and had a relatively small sample size, suggesting the need for larger, more robust trials to confirm these findings [49].

Further supporting the role of alemtuzumab in high-risk populations, a review of its clinical pharmacokinetics and pharmacodynamics shows that while it effectively reduces acute rejection, it also increases the risk of complications, including infections such as cytomegalovirus (CMV), and may lead to de novo donor-specific antibodies (DSAs), which are associated with chronic graft dysfunction. These effects underline the importance of close post-transplant monitoring and personalized infection prophylaxis for patients receiving alemtuzumab [50].

Given these findings, more research is needed to fully understand alemtuzumab’s long-term effects, particularly in high-risk populations like Black recipients. While studies like Hanaway et al. have shown that alemtuzumab can improve short-term outcomes in high-risk transplant recipients, further investigation is required to assess its impact on long-term graft survival and patient safety [51].

## Conclusion

This review underscores the complexity and necessity of personalized induction therapy in kidney transplantation, particularly for high-risk groups such as Black patients and those with donor-specific antibodies (DSAs). The evaluation of the NICE, KDIGO, and ERBP guidelines reveals significant strengths and limitations in their approaches to induction therapy.

The NICE guidelines offer a standardized approach, recommending basiliximab as the preferred agent across a broad spectrum of transplant scenarios. However, their broad application may not adequately address the unique needs of high-risk patients, such as those of Black ethnicity, who often exhibit heightened immunological responses and require more potent induction therapies like lymphocyte-depleting agents.

In contrast, the KDIGO and ERBP guidelines advocate for a more personalized approach, recognizing the importance of tailoring immunosuppressive regimens based on individual genetic and immunological profiles. These guidelines emphasize the use of potent agents such as anti-thymocyte globulin (ATG) or alemtuzumab for high-risk groups, aligning with the growing body of evidence supporting personalized medicine.

A critical finding of this review is the lack of a clear, genetics-based definition of Black ethnicity in the existing guidelines. Black patients face higher immunological risks due to factors such as HLA diversity and faster metabolism of immunosuppressive drugs, necessitating more tailored therapeutic strategies. Genetic markers like HLA alleles, CYP3A5, and APOL1 variants play crucial roles in determining the appropriate induction therapy, underscoring the need for genetic screening and personalized dosing regimens. Furthermore, the review highlights the importance of genetic testing and population studies in understanding the genetic diversity within Black populations. Implementing genetic insights into clinical practice can significantly enhance the precision and effectiveness of medical treatments, reducing health disparities and improving transplant outcomes. Advocating for personalized induction therapy is essential, considering the unique genetic and immunological profiles of each patient. This approach not only optimizes transplant success and minimizes adverse effects but also ensures equity in healthcare by addressing the specific needs of racially diverse populations.

In conclusion, the development of a more nuanced and effective framework for induction therapy in kidney transplantation must recognize the complexities of high-risk groups and integrate personalized medicine principles. By tailoring induction therapies to the genetic and immunological profiles of transplant recipients, healthcare providers can improve clinical outcomes and promote equity in transplantation medicine.

## Data Availability

All data that support this study has been provided and are also available on request from the corresponding author.
